# Alignment of Selected Veterinary Education Competencies With the Interprofessional Professionalism Assessment

**DOI:** 10.3389/fvets.2021.688633

**Published:** 2021-07-09

**Authors:** John H. Tegzes, Jody S. Frost

**Affiliations:** ^1^Office of Mission Integration, College of Veterinary Medicine, Western University of Health Sciences, Pomona, CA, United States; ^2^National Academies of Practice, Lusby, MD, United States

**Keywords:** interprofessional, professionalism, competency-based education, assessment, Entrustable Professional Activities (EPAs)

## Introduction

The move toward outcomes and competency-based veterinary education began decades ago but has accelerated in the past few years. While many advances in curriculum structure and delivery are replete in the literature, assessment methods remain challenging and less explored. This is especially true for competency domains that are behavior-based such as professionalism, which has been challenging to merely define let alone assess. The Interprofessionalism Professionalism Assessment (IPA) is a validated tool that is used in veterinary education and is useful in assessing competency domains that have remained challenging for educators. Here we present the challenges and solutions for assessing competency in veterinary education and provide examples and data from tools that may be useful especially in areas such as professionalism.

## Background

It is important to consider the pathway that veterinary education has traveled toward competency-based education and assessment before diving into specific assessment methods. This section will provide a brief overview of the history of outcomes-based and competency-based education as it applies to veterinary education.

### Outcomes-Based Education

Outcomes-based education was initiated in 1949 when educational psychologist Ralph Tyler presented four questions to educational institutions now known as the “Tyler rationale” (1, 2). At the time, the questions were a sharp contrast to how educators organized or conceptualized their work. Through these questions, he ushered in the idea that education should be driven by outcomes, asking:

1. “1 What educational purposes should the school seek to attain?
2. How can learning experiences be selected which are likely to be useful in attaining these objectives?
3. How can learning experiences be organized for effective instruction?
4. How can the effectiveness of learning experiences be evaluated?”

These questions have led to subsequent frameworks to evaluate teaching and learning. Educational researchers have added wisdom over the years. Many have been adopted as a matter of routine in contemporary education, whether in primary or in health professions education. Educators commonly use Bloom's taxonomy for describing the progression of learning in three domains: cognitive, psychomotor, and affective (3). Kirkpatrick's framework is often used to measure the effectiveness of curricular methods (4). Based on these frameworks, it might seem predictable that the next evolution in education would be teaching for competence.

### Competency-Based Education

Competency-based education embraces outcomes-based learning, advancing the framework to another level, especially when applied to healthcare professions' education. It is organized around competencies, or specific abilities, as the outcomes of the curriculum (5). In 1978, McGaghie et al. (5) described two alternative curriculum models in medical education that could replace the widely accepted subject-centered structure of most curricula of the time. The authors described the first as an integrated program where learning and teaching fuse formerly separate medical disciplines by using organ systems or medical problems as the organizing structure. The second model described was one that focuses on the functional elements of medical practice where the emphasis is on learning how to practice medicine as opposed to accumulating knowledge about medical practice. The authors called this “competency-based” because the emphasis was on learning how to practice medicine rather than on the accumulation of knowledge (6, 7).

This evolution from educating for knowledge to educating for competency seems logical, but also poses many questions without necessarily obvious answers. For instance, what is meant by competence? How can it be measured? Rather than highlighting a specific skill or procedure, competence is measured by comprehensive performing. Caring for a patient involves more than a specific skill or even set of skills. It requires knowledge, clinical expertise, and human connectedness demonstrated as behaviors (7). As competency-based education has been defined it leads to a primary focus of education on the desired outcomes for learners rather than on the structure of individual courses (8). Competence is an amalgamation of taking what was learned in the classroom, what was learned through independent study and reading, practicing specific clinical skills, communicating, working in a team, and finally reflecting upon all of these elements and modifying performance. Integrating all of these components within a specific context enables one to practice a specific healthcare profession (7). While at first glance it might seem like a practicing physician, dentist, or veterinarian might need to demonstrate competence while caring for many different types of patients with various diseases, the competency frameworks are quite short and non-specific, making it all the more difficult to assess for competency. Not surprisingly it would explain why educators may struggle with formulating specific assessments, and why sometimes you may hear the phrase, “I know competence when I see it” before being able to provide an explicit definition.

### Competency-Based Frameworks

In Canada, frameworks for identifying and defining competence in medical education were initially sparked by calls in the 1990s for physician accountability and professionalism (7). CanMEDS defined a framework of competencies designed to address the roles physicians have in meeting societal needs. The CanMEDS Framework identified the competencies of physicians, which included medical expert, communicator, collaborator, manager, health advocate, scholar, and professional (7). Today there are similar frameworks in the United States, the United Kingdom, and in The Netherlands (2, 6). According to ten Cate (2) these competency frameworks contain logical sets of qualities that every physician should acquire and are still a theoretical construct. The defined competencies are general attributes of a good doctor. Unfortunately, as we begin to assess competencies in educational settings, they tend to get reduced to a detailed list of skills. Often the skills most emphasized are those that are procedural with a specific endpoint that can be measured for success (i.e., inserting a thoracostomy tube). Educators tend to focus on skills and activities that are objective, measurable, and repeatable (reproducible). This creates dissent and debate among educators when competencies have titles such as collaborator and professional which are often measured somewhat subjectively and contextually in the clinical settings.

As competency frameworks in medical education have been adopted across various countries, so too have they spread to other professions (9–12). The existing competency frameworks cannot simply be applied to the profession of veterinary medicine without revision and adaptation. While educating physicians and veterinarians share many similarities, there are also clear differences. For instance, most physicians must undergo further post-graduate education in the form of residency programs before becoming licensed for practice. Most veterinary students must be practice-ready on day 1 following graduation, with residency and fellowship programs remaining optional, and occurring after full licensure. Therefore, their readiness for practice upon graduation is paramount and different. Much work has been done to establish a competency-based framework for veterinary education. In The Netherlands, the work of Bok et al. paved the early road to identifying competencies in veterinary education. The authors established what was referred to as the Veterinary Professional, or VetPro framework (13).

The VetPro Framework was established in consultation with practicing veterinarians, and later validated internationally among practicing veterinarians. Veterinarians with clinical experience across many species groups were consulted in order to identify the core competency domains necessary for the practice of veterinary medicine across all typical practice settings. A total of seven competency domains were identified and defined. These included communication, personal development, collaboration, entrepreneurship, veterinary expertise, health and welfare, and scholarship (13). After the initial work that identified the competency domains was conducted in The Netherlands, an international survey was conducted to explore whether there was international consensus on the competency domains. Veterinarians in The Netherlands, Spain, Norway, United States, South Africa, Switzerland, Canada, United Kingdom, Malaysia, and Australia were included. While there was overall agreement with the importance of veterinary expertise as a competency domain, there were some differences in the importance of the other competency domains, although all were considered important to a degree (14).

In 2015, the Competency-Based Veterinary Education (CBVE) Working Group was established to develop an internationally shared framework and assessment tools for use in veterinary education. The goal was to form a unified and comprehensive competency framework that reflects expectations of newly graduated veterinarians, guides learner assessment, and promotes targeted curricular outcomes assessment (15). Likewise, it was the intent that this framework would guide veterinary educational institutions in implementing competency-based methods of instruction. Nine competency domains were established as well as eight core Entrustable Professional Activities (EPAs) linked to the framework (15). An EPA is defined as an essential task that an individual can be trusted to perform without direct supervision in a given health care context, once sufficient competence has been demonstrated (16). To help learners and assessors monitor progression toward competence, milestones are often established within each EPA. Milestones are defined as observable markers of an individual's ability along a developmental continuum (16). Milestones for these veterinary EPAs have also been developed (17). The core competency domains established for veterinary education include (15):

1. Clinical Reasoning and Decision-making
2. Individual Animal Care and Management
3. Animal Population Care and Management
4. Public Health
5. Communication
6. Collaboration
7. Professionalism and Professional Identity
8. Financial and Practice Management
9. Scholarship

The eight EPAs established include (18):

1. Gather a history, perform an examination, and create a prioritized differential diagnosis list
2. Develop a diagnostic plan and interpret results
3. Develop and implement a management/treatment plan
4. Recognize a patient requiring urgent or emergent care and initiate evaluation and management
5. Formulate relevant questions and retrieve evidence to advance care
6. Perform a common surgical procedure on a stable patient, including pre-operative and post-operative management
7. Perform general anesthesia and recovery of a stable patient including monitoring and support
8. Formulate recommendations for preventive healthcare.

### Clinical Workplace-Based Assessments

**Figure 1:**
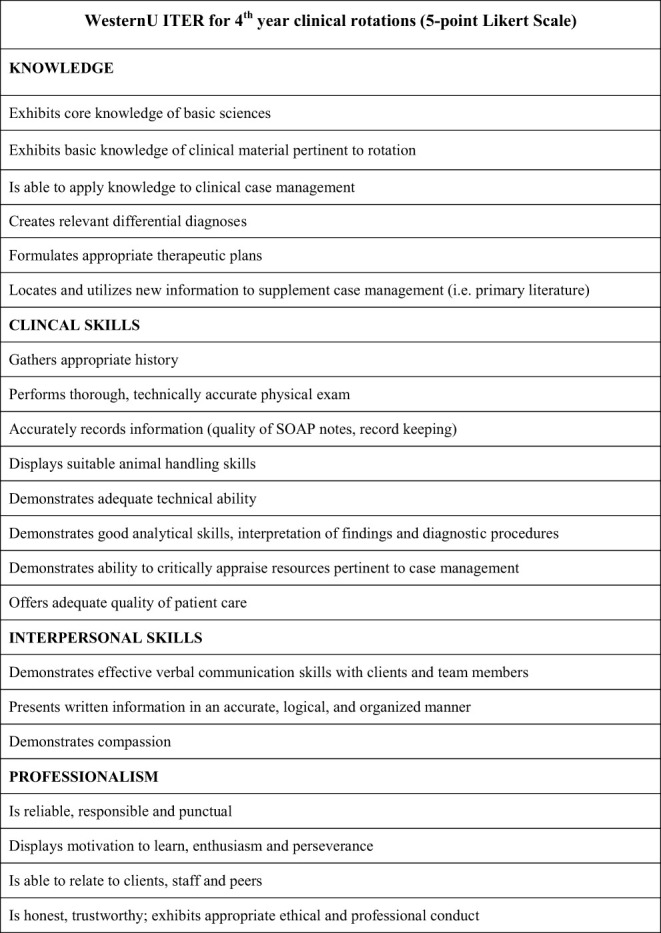
Western University of Health Sciences ITER used during 4th year clinical rotations.

As is evident from the list of EPAs, the clinical workplace is the environment generally where demonstration of progress and assessment of entrustability can occur. EPAs typically span across multiple competency domains. The milestones provide a shared mental model for how learners are expected to developmentally progress throughout the program in providing a roadmap to building competence. Given that each EPA spans multiple competency domains, then a variety of assessment tools can be useful. Traditionally, in-training evaluation report scales (ITERs) have been used in veterinary education (19). They are usually completed by clinical preceptors who observe students throughout a clinical rotation. These rotations can span various time periods with two-to-four-week rotations common across veterinary institutions and are usually completed once at the end of the rotation. Rather than giving feedback about a particular moment in time, or a unique clinical encounter, they provide an assessment of the overall performance over the entire clinical rotation typically using a Likert-scale type of tool. They can encompass areas such as knowledge, clinical skills, interpersonal skills, and professionalism (see Figure 1). They are sometimes customized to fit the clinical environment where they are being used by clinical preceptors and faculty (20). Often, they require training and calibration of evaluators. For institutions that frequently use a large number of clinical preceptors and evaluators this can become problematic. Even when time is taken for calibration and instruction consistency of use can still be lacking as they are sometimes recognized by learners as “staff-dependent” (21). Variability between assessors, however, can be seen as providing valuable formative feedback for learners. Still, as EPAs are established in veterinary education the search for psychometrically sound and useful assessment tools will progress. Importantly, whatever tools are used need to not only assess entrustability, but also to provide formative feedback that can be used to guide learner progress, especially in areas like professionalism that remain somewhat ambiguous or subjective to many learners. As others have previously noted, clinical workplace-based assessments and entrustment scales can provide formative information and feedback to learners and help to determine when learners are able to engage in independent clinical practice (19). This formative process is essential to building competence and entrustability.

Clinical workplace-based assessments can be used strategically to steer learning toward desired outcomes, especially when formative timely feedback is provided during and after the assessment event (22). The form of the feedback can be both verbal and written. In addition to ITERs, there are other methods that have shown positive impacts on learning, including the Mini Clinical Evaluation Exercise (Mini-CEX) and the Direct Observation of Procedural Skills (DOPS) (23).

The Mini-CEX is used to evaluate learners while performing clinical tasks that often require demonstration of multiple skills simultaneously, such as taking a history while performing a physical examination of a patient. The core purpose is to provide structured feedback based on observed performance (22). Likert scaled scoring forms are often used that provide feedback not only on skills, such as physical examination, but also on communication and professionalism.

The DOPS assessment method focuses on evaluating procedural skills performed in the clinical workplace. Learners are provided a list of commonly performed procedures for which they are expected to demonstrate proficiency, and that are assessed by multiple clinicians on numerous occasions during an educational period of time (22). As with the Mini-CEX, the main goal of the DOPS is the provision of structured feedback based on observation of performance rather than on a simple completion log of procedures performed (22). The goal is to improve skills while performing specific procedures with the emphasis on the procedures themselves without much feedback given for competencies such as communication or professionalism.

While these three assessment and feedback instruments might sound similar, they do provide different information to learners. Take for instance a clinical scenario wherein a learner must successfully perform venipuncture on a dog after taking a history from the client and performing a physical examination. It is possible that proficiency could be marked as below expectations, meets expectations, or even exceeds expectations for the exact same clinical encounter based on the assessment tool used. Imagine that a veterinary student is given that specific task; to take a clinical history from a client, perform a physical examination, and to successfully draw blood from the patient, then divide the blood sample into the appropriate diagnostic collection tubes. During the encounter, the student only asks closed-ended questions and fails to elicit an important detail about the animal's clinical history. The student performs the physical exam adequately and note the appropriate abnormalities both verbally and in writing afterwards. During the venipuncture the student fails to communicate with the veterinary technician the proper restraint technique required and the client, who is standing close-by, gets bitten by their own dog. The learner, however, manages to collect the venipuncture sample and divides the sample appropriately into red-top and lavender-top tubes. If safety issues are incorporated into an ITER it would likely capture the obvious safety issue with improper restraint and injury to the client, resulting in a below-expectation score for the encounter even though the evaluation is a composite of the entire rotation because it would likely stand out in the preceptor's mind as a concern. A DOPS, however, would likely only note the successful collection and dividing of the blood samples, rating the event as meets or exceeds expectations. A Mini-CEX would provide feedback on the technical skills, communication, and collaboration with the technician, with ratings denoted as mixed results. Therefore, the tools used in the clinical workplace can make a profound difference in the focus of the feedback provided. This is important to recognize as EPAs span multiple competency domains and invites the discussion about whether multiple unique tools should be used when evaluating learning and providing formative feedback.

It has been proposed by veterinary educators and researchers to restructure veterinary education with EPAs (24). There is evidence that students are able to gradually gain experience in EPAs with various members of the clinical team (25). Using EPAs in the clinical workplace is a viable way to recognize student work and guide the development and progression of learners across the continuum of the various principles underlying them (26). Yet, whether in medical, veterinary, or other healthcare professions education, some of the competency domains can be more challenging to assess. Communication, collaboration, and professionalism are contextual and challenging to merely affirm that a learner was effective. Professionalism is a competency that some will say, “they know it when they see it,” yet can be difficult to describe let alone create a scoring rubric. The IPA is a validated tool that is used by various members of the clinical team and provides formative feedback and evaluates various aspects of professionalism that are not readily captured by other evaluation tools. It has been used in veterinary education in both classroom and clinical settings to assess behaviors associated with professionalism in an interprofessional context. In the context of EPAs, the IPA could be used to help clinical educators and evaluators provide both formative and summative feedback to learners in domains difficult to assess with traditional tools. It could be used alongside these traditional tools by emphasizing areas that are typically missed with them, thereby providing a more holistic view and approach to clinical assessment. The real danger of not having specific tools to measure specific elements of EPAs is that some competencies may be merely passed over when tools emphasize only the more tangible hands-on skills. Worse still, learners may be given a “pass” on professionalism skills when they are condensed into other clinical skills performed at the time. The development of the IPA began with establishing a formal definition of interprofessional professionalism, which is stated as, “the consistent demonstration of core values evidenced by professionals working together, aspiring to, and wisely applying principles of altruism and caring, excellence, ethics, respect, communication, and accountability to achieve optimal health and wellness of individuals and communities” (27, 28).

### Interprofessional Professional Assessment (IPA)

The IPA was created over a 9-year period through extensive development and pilot testing by the Interprofessional Professionalism Collaborative (IPC), a national organization with representatives currently from 12 entry-level health professions and the National Board of Medical Examiners. The IPA instrument is a 26-item observational rating tool used by faculty and preceptors to assess learners' professionalism when working with members of other health professions. The tool was piloted at the end of a practice experience (e.g., rotation) in environments where interprofessional, collaborative care of patients was conducted. The psychometric properties of the IPA were tested with preceptors from 10 different health professions, including veterinary medicine, in seeking to support its generalizability. Psychometric results demonstrate aspects of IPA reliability and validity, and its use across multiple health professions and in various practice sites (27–29).

The development of the IPA was comprehensive and well-executed from 2006 to 2015 over three phases: (1) Construct development and generation of observable behaviors and response scales (27); (2) Content expert review and cognitive interviews with typical raters (27, 28); and (3) a 2-year pilot study (29). The process began with a literature review, construct definition, and the organization of 200 potential behaviors into categories by the members of the IPC. The number of behaviors was reduced to 43 after the IPC applied explicit inclusion criteria (e.g., behaviors that are positively oriented and observable in practice, applicable across multiple professions, not redundant). Members of the IPC then made national and international presentations about the tool, documented oral feedback from audience members, and collected follow-up online survey feedback from 205 individuals representing 11 professions. This feedback led to the formatting of a 39-item instrument, which was then reviewed by a panel of 23 content expert reviewers from the U.S. and Canada. The panel responded to structured survey questions about the tool's content, fit of 39 behavioral items within and across six domains, overall organization, format, and length (28).

**Figure 2:**
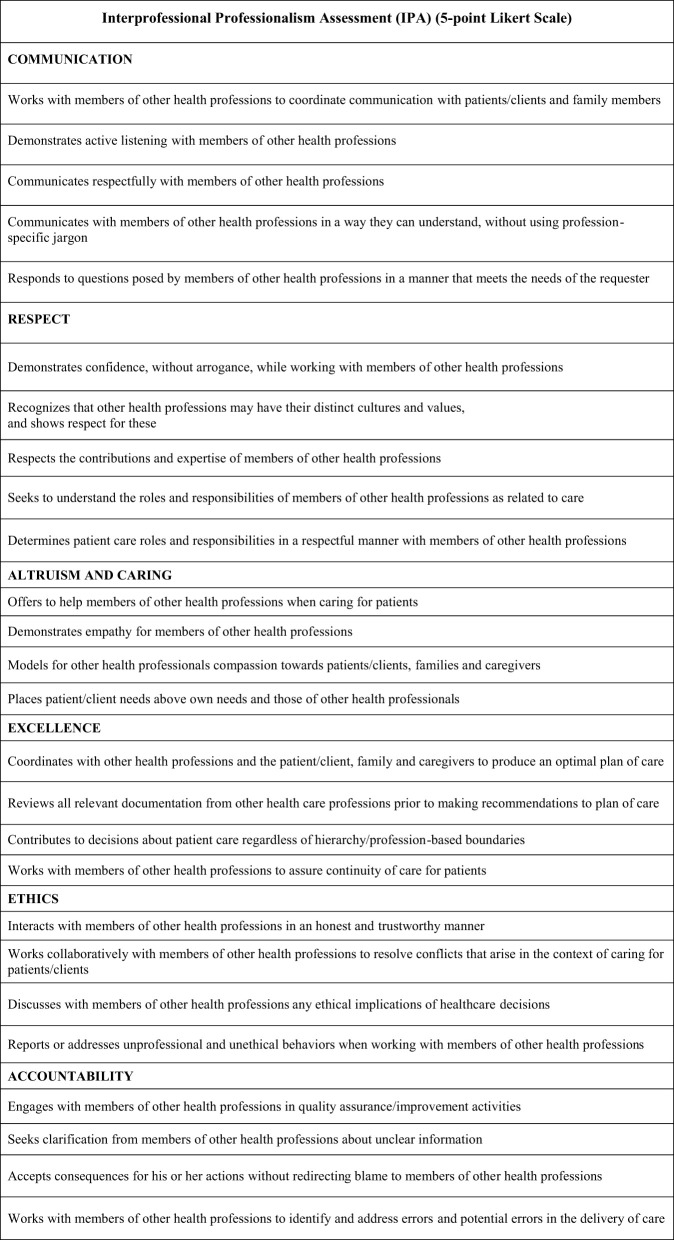
Interprofessional Professionalism Assessment (IPA).

Twenty-four preceptors, two from each of the 12 IPC member health professions representing “typical” preceptors that would use the tool, were involved in two rounds of cognitive interviews. Based on their feedback, the IPA was reduced to 26 items categorized into six competency domains which included (see Figure 2):

1. Communication
2. Respect
3. Altruism and Caring
4. Excellence
5. Ethics
6. Accountability

The 26 behavioral items are constructed on a 5-point, Likert-type scale with the following anchors: 1 = “strongly disagree,” 2 = “disagree,” 3 = “neutral,” 4 = “agree,” and 5 = “strongly agree.” For each behavior there is also the response option, “no opportunity to observe.” The two qualitative items provide space for raters to comment on the “overall strengths related to interprofessional professionalism” and “areas for improvement related to interprofessional professionalism” (28).

The 26-item instrument is the version of the IPA which was used in a large, multi-institution and multi-profession pilot study. A total of 67 academic institutions were invited to participate in the pilot; 30 agreed to do so (44.8%). Using a key contact method, nearly 3,000 preceptors (estimated) were invited into the study; 376 agreed and 233 provided data (62% of enrolled, 7.9% of potential population) (29).

Exploratory factor analysis (EFA) was conducted on preceptors' ratings of their learners assuming ordered categorical factor indicators. To determine the number of factors to retain, eigenvalues and measures of fit were examined. Prior to factor analysis, the extent of missing data for each of the IPA items was examined (i.e., an item either left blank or the respondent recorded N/O – No opportunity to observe in this environment). Internal consistency reliability of the factors suggested by the EFA was calculated using coefficient alpha. The initial EFA using 21 items (excluding five items with extensive missing data) suggested retaining four factors. With eigenvalues of 12.670, 1.229, 0.888, and 0.787, the four factors together accounted for 86.5% of the variance in the set of variables, and the fit indices indicated good model fit (RMSEA = 0.064, 90% CI: 0.055–0.078; CFI = 0.991; SRMR = 0.027). The four factors loaded well on the following domains: Communication, Respect, Excellence, Altruism, and Caring. Internal reliability consistency coefficients were high (alpha >0.94) for each of the factors. Despite the psychometric results, and based on other considerations, the study authors decided to keep the 5 excluded items and the two other domains (Ethics and Accountability) in the final instrument (29).

During the pilot study, responding preceptors were also asked to complete two global items for each learner they evaluated: one was a global rating of the learner's interprofessional professionalism, and the other a global rating of the learner's overall performance on the practice experience. These ratings were made using a 5-point Likert-type scale (1 = “poor,” 5 = “excellent”). Given the results of the factor analysis, items within each domain were averaged to create subscale scores and factor scores were also estimated from the final factor model. These scores were all positively and significantly correlated with the two global performance items described above (29, 30).

The Interprofessional Professionalism Collaborative (IPC) has a website (http://www.interprofessionalprofessionalism.org/assessment.html) from which a PDF of the IPA instrument can be downloaded for use. The IPC website also has a toolkit which provides training videos for users (e.g., students, faculty, preceptors) and narrative written and recorded scenarios to support training in the use and application of the IPA in their practices and programs.

## Discussion

The introduction of competency-based education in veterinary medicine requires not only a change in how we educate, but perhaps more importantly a change in how we assess learners. Historically, assessments in the final clinical years of veterinary education have been less formalized than written assessments in the pre-clinical years. But with an emphasis on competence and entrustability, assessment in the clinical years is perhaps more important than traditional multiple-choice types of exams during the pre-clinical years. While there has been tremendous progress in defining competence, and describing competence-based education, many still struggle with methods of assessment that are valid and reliable. Workplace-based assessments have been studied and examined more extensively in medical education than in veterinary education. Yet, one must be careful to accept validation only in medical education and expecting similar applicability to veterinary education. Therefore, there is extreme importance in validating instruments in the veterinary setting and amending those with re-validation, as applicable.

The IPA is an example of an assessment tool that has been studied and validated in the veterinary setting (29). As an interprofessional assessment tool, it is important to note that interactions with other health professions are not frequent in veterinary practice. A study examining the frequency of interactions between veterinarians and other healthcare providers found that interactions with pharmacists was the most common interprofessional interaction (31). In the study and validation of the IPA, a total of 10 professions, including veterinary medicine, were included (29). The ratings on the IPA for veterinary interprofessional interactions were scored similarly to interactions that did not include a veterinary component. Veterinary students at Western University of Health Sciences, College of Veterinary Medicine (WesternU CVM) were participants in the IPA validation study. It is important to note that the WesternU CVM utilizes a distributive model of clinical education as opposed to a standing university teaching clinic. As such, many clinical rotations occur in the greater Los Angeles metropolitan area where interactions with other health professions were most likely to be with pharmacists and in some instances with physical therapists in animal rehabilitation settings.

While the design and validation of the IPA tool was not originally intended for intra-professional interactions, the IPA tool does have applicability when evaluating interactions with veterinary technicians, animal assistants, farriers, and others within the broader field of veterinary practice. A major impetus for interprofessional education and collaborative practice efforts has been to improve patient outcomes and safety (32–34). The Institute of Medicine reports have emphasized how patients are harmed when communication and collaboration fail in healthcare. Failures in communication and collaboration have been reported as preventable causes of death and injury in healthcare settings (32). While it is generally unclear if the same deleterious effects occur in the veterinary setting when communication and collaboration fail, there are indications that the patterns may be similar (35). Further, substantial barriers may exist for reporting significant events in veterinary practice (36). The reasons for under-reporting are often attributes and behaviors that are captured in the IPA tool, specifically under the competency heading of accountability. There is a clear need to better assess professionalism behaviors in veterinary learners not only to improve the quality and safety of care and thus patient outcomes, but also to promote effective collaboration within the veterinary team, and to improve relationship-centered care with veterinary clients.

Increasingly, veterinarians are being employed outside of the historic, traditional veterinary practice owned by one or a few of the veterinarians working there. Corporate veterinary practices are expanding across the entire US. Additionally, specialty practices are expanding in both the companion animal and production animal veterinary markets. The idea of the veterinary team consisting of a group of people working under the same roof, with similar working hours is being replaced by primary care and specialty care teams working across geographic locations in multiple practices operating 24 hours a day and 365 days a week. Teamwork skills have never been needed more in veterinary medicine in order to maintain the quality of care and ensure the safety of patients and the public. Veterinary careers also are expanding in research and biotechnology organizations, in public health sectors, in non-profit organizations, and in government. With these expansions the veterinary team is changing. Clinical veterinary teams may include specialists working under the umbrella of a corporate veterinary practice with locations spanning several cities or states across the country. New veterinary specialty organizations are in the works, with further expansions of specialty care being defined. Outside of clinical practice, veterinary teams might include epidemiologists and environmental scientists working in public health, policymakers and lawmakers working in government, and engineers and computer scientists working in industry to name a few, where professionalism takes on a broader perspective and includes new stakeholders. The need to train veterinarians and veterinary students in professionalism behaviors and effective teamwork practices and skills has never been greater. Along with the need to educate comes the even greater need to reliably assess such learning. Not only do professionalism behaviors need to be consistently defined, they must also be reliably assessed wherever veterinary teams are working. And this is reflected in the competency domains identified and defined by work done by the CBVE.

The IPA presents many opportunities to discuss attributes and behaviors associated with professionalism with veterinary students, throughout the professional veterinary educational program. At WesternU it has been used successfully with pre-clinical years students in formalized interprofessional education courses that include veterinary students. With students in the pre-clinical years, it is not possible to use the tool in the workplace-based setting. Instead, filmed interprofessional interactions (scripted) are used. To begin, information is provided to students about the IPA tool and initially introduced through an assigned reading, then reviewed in the classroom setting. Application of the IPA is demonstrated by showing a filmed interprofessional interaction. Each student is assigned a clinician (actor) to review using the IPA tool. They first watch the filmed interaction and are instructed to take notes on what they observe. Next, they are provided portions of the IPA tool to review and instructed to watch the same video a second time. This time they are asked to complete the IPA portions applicable to the scene. Students complete this activity as individuals without first comparing their reviews. Later they compare their evaluations with those of their peers and discuss how they arrived at their scores, noting specific behaviors that they observed that support the ratings. Finally, students discuss how and why multiple people watching the same interactions might score them differently. The scripted scenes used were carefully chosen because the words that the actors use do not always reflect the intentions in their actions. It provides rich opportunities for students to discuss the power of both verbal and non-verbal communication, and the implications when collaboration and communication fail. During the debrief of the activity, the discussion often includes hierarchical issues in healthcare that impact how individuals within certain professions behave and misbehave in the clinical environment. It is hoped that through these classroom activities, students are sensitized to notice communication successes and failures when in the clinical workplace environments in subsequent years of the curriculum.

## Current and Future Work

While the IPA was developed using the traditional definition of interprofessional education which includes learners from different healthcare professions learning with, from, and about one another in order to improve collaboration and the quality of care, it has value when applied to the intraprofessional veterinary team (37). The typical clinical veterinary healthcare team includes veterinarians, veterinary technicians or nurses, animal assistants, and others depending on the practice environment. With the expansion of specialty practices, especially in major urban centers, the veterinary team can include veterinary specialists and primary care clinicians working collaboratively with the same patients but in different practice settings. Often these collaborations occur across distant practice settings and not within the same hospital, as is common within human healthcare hospitals and clinics. When communication fails, collaboration is not promoted, and respect is absent or lacking, there may be a failure to deliver optimal care for a given situation, or harm may come to the veterinary patient (35, 38). In a study examining errors in veterinary practices, many of the root causes were aligned with communication failures (both verbal and health record omissions), and failures in team functioning (39). These errors often involved failures in collaboration among veterinarians, veterinary nurses, and receptionists. Failures in communication may ultimately become a potential legal liability; indeed, failures in communication and deficiencies in interpersonal skills were linked to complaints against veterinarians filed with state veterinary boards (40). Additionally, the presence or absence of these skills appear to impact professional well-being and satisfaction. A primary reason for veterinary nurses leaving the profession was found to be “lack of respect and recognition from veterinarians” (39).

A core tenet of interprofessionalism involves placing the patient and client at the center of the healthcare team. In the veterinary setting this translates to client-centeredness. The veterinary client hierarchy of needs suggested by Hughes et al. (41) focuses on working in partnership with clients, and emphasizes the importance of communication skills, professionalism, clinical problem-solving, and the animal's welfare in achieving excellence. These aspects are captured well with the IPA, and therefore it is also applicable to apply its use while evaluating veterinary students' interactions with clients.

Interprofessional learning within the veterinary healthcare team has been successfully piloted and demonstrated to change attitudes, overcome misconceptions about the professions and promote the importance of communication between veterinarians and veterinary nurses (42). Veterinary students also participate in traditional interprofessional education programs, and benefit from learning with, from and about students in the human healthcare professions (43). The IPA has been validated and used in the veterinary education setting and can be applied and used in the education of both veterinarians and veterinary technicians/nurses. Therefore, more work could be done evaluating its merits within existing veterinary teams, and while educating both veterinary students and veterinary technicians, whose educational programs tend to be unconnected and isolated even though they work very closely together after graduation.

In addition to traditional veterinary teams working in typical practice locations, the veterinary team also includes those working in academia, government, public health, biotechnology, and industry among others. The IPA has applicability in these teams as well with very minor modifications such as replacing “patient” with “stakeholder” for example. The importance of professionalism does not stop at clinical locations or at the cage-side. Future work might include these expanded veterinary roles working in teams that are indeed interprofessional, but not necessarily with the professions that usually come to mind when thinking only of the clinical setting.

Other areas of future work could be in comparing use and applicability in urban vs. rural settings, where the needs of clients and stakeholders might be perceived differently. While the needs might not be that different in actuality, the culture within an organization may impact how professionalism behaviors are demonstrated and addressed. Therefore, examining the IPA tool in various urban and rural locations might help to provide important data.

Lastly, improving diversity within the veterinary profession and within veterinary teams is important and a focus of national and international attention. While the IPA tool was investigated in a large-scale pilot it was not examined for applicability of use with various ethnic groups, races, ages, gender, or gender identity. Designing studies to look at its applicability across these and other social constructs is warranted.

## Author Contributions

All authors listed have made a substantial, direct and intellectual contribution to the work, and approved it for publication.

## Conflict of Interest

The authors declare that the research was conducted in the absence of any commercial or financial relationships that could be construed as a potential conflict of interest.
